# Matrix Expansion and Syncytial Aggregation of Syndecan-1^+^ Cells Underpin Villous Atrophy in Coeliac Disease

**DOI:** 10.1371/journal.pone.0106005

**Published:** 2014-09-08

**Authors:** Camilla Salvestrini, Mark Lucas, Paolo Lionetti, Franco Torrente, Sean James, Alan D. Phillips, Simon H. Murch

**Affiliations:** 1 Department of Paediatric Gastroenterology, Addenbrooke's Hospital, Cambridge, United Kingdom; 2 Centre for Paediatric Gastroenterology, University College London, United Kingdom; 3 Department of Paediatrics, University of Florence, Meyer Hospital, Florence, Italy; 4 Department of Pathology, University Hospital Coventry & Warwickshire, Coventry, United Kingdom; 5 Division of Metabolic and Vascular Health, Warwick Medical School, Coventry, United Kingdom; Charité, Campus Benjamin Franklin, Germany

## Abstract

**Background:**

We studied the expression of sulphated glycosaminoglycans (GAGs) in coeliac disease (CD) mucosa, as they are critical determinants of tissue volume, which increases in active disease. We also examined mucosal expression of IL-6, which stimulates excess GAG synthesis in disorders such as Grave's ophthalmopathy.

**Methods:**

We stained archival jejunal biopsies from 5 children with CD at diagnosis, on gluten-free diet and challenge for sulphated GAGs. We then examined duodenal biopsies from 9 children with CD compared to 9 histological normal controls, staining for sulphated GAGs, heparan sulphate proteoglycans (HSPG), short-chain HSPG (Δ-HSPG) and the proteoglycan syndecan-1 (CD138), which is expressed on epithelium and plasma cells. We confirmed findings with a second monoclonal in another 12 coeliac children. We determined mucosal IL-6 expression by immunohistochemistry and PCR in 9 further cases and controls, and used quantitative real time PCR for other Th17 pathway cytokines in an additional 10 cases and controls.

**Results:**

In CD, HSPG expression was lost in the epithelial compartment but contrastingly maintained within an expanded lamina propria. Within the upper lamina propria, clusters of syndecan-1+ plasma cells formed extensive syncytial sheets, comprising adherent plasma cells, lysed cells with punctate cytoplasmic staining and shed syndecan ectodomains. A dense infiltrate of IL-6^+^ mononuclear cells was detected in active coeliac disease, also localised to the upper lamina propria, with significantly increased mRNA expression of IL-6 and IL-17A but not IL-23 p19.

**Conclusions:**

Matrix expansion, through syndecan-1+ cell recruitment and lamina propria GAG increase, underpins villous atrophy in coeliac disease. The syndecan-1+ cell syncytia and excess GAG production recapitulate elements of the invertebrate encapsulation reaction, itself dependent on insect transglutaminase and glutaminated early response proteins. As in other matrix expansion disorders, IL-6 is upregulated and represents a logical target for immunotherapy in patients with coeliac disease refractory to gluten-free diet.

## Background

In coeliac disease (CD), distinct architectural changes occur which reduce the absorptive surface. The characteristic lesion of active CD consists of villous atrophy, crypt cell hyperplasia and marked lamina propria leukocyte infiltration [1]. The term villous atrophy is misleading, since the volume of the lamina propria is doubled [2]. Two classic studies of the coeliac mucosal lesion suggest that pathological matrix expansion may occur. Three-dimensional morphometry by Loehry and Creamer showed that villous atrophy occurs due to expansion of intervillous lamina propria, akin to a cake rising during cooking [3]. Shiner identified electron-microscopic changes in the subepithelial lamina propria within 24 hours of gluten challenge, characterised by increase in fibrillar connective tissue together with dense recruitment of plasma cells, many showing evidence of lysis [4]. Thus villous atrophy in coeliac disease occurs primarily due to matrix expansion rather than villous shortening.

The volume of tissues is largely determined by expansion, within a meshwork of inexpansible fibrils, of a gel matrix composed of hydrated negatively-charged GAGs, which maintain tissue turgor through charge-based sequestration of albumin and electrolytes [5]. In addition, the strongly anionic heparan sulphate proteoglycans (HSPG) play multiple roles in physiology through specific interaction with numerous cytokines and receptors [6]. Within the intestine, epithelial HSPG critically limit transepithelial protein leak, and their loss promotes protein losing enteropathy (PLE), in synergy with inflammatory cytokines and hydrostatic pressure [7],[8].

Tissue swelling due to cell recruitment and matrix expansion is a common pathological event which has received relatively little attention. Disorders characterised by extracellular matrix expansion and excess GAG production include proptosis and acropachy in autoimmune thyroid disease [9], facial swelling in lepromatous leprosy [10], many causes of hepatomegaly [11] and chronic asthma [12]. The mechanisms of tissue expansion include recruitment of inflammatory cell subsets and a stimulated hyperproduction of GAGs by fibroblasts and other cells. Such a combination of inflammatory cell recruitment and stimulated GAG production is also seen in the invertebrate encapsulation reaction, first noted by Metchnikoff in starfish larvae, in which recruited haemocytes palisade around invading pathogens too large to phagocytose: production of a wall of GAGs by these cells effectively isolate the invader and deprive it of nutrients [13]–[15]. The questions thus arise whether stimulated matrix expansion represents an evolutionarily conserved cell-mediated defense mechanism and whether there may be commonality of mechanism in different diseases (including coeliac disease) characterised by induced matrix expansion.

Increased expression of various potentially matrix-shaping cytokines, including IFN-γ, TNF-α, and IL-6 has been reported in coeliac disease [1],[16]. Amongst candidate cytokines, IL-6 has particularly strong credentials as a mediator of physiological or pathological matrix expansion, inducing GAG production amongst various cell types and in diverse disease states, including Grave's disease and gingival hyperplasia [17]–[19], while hepatic regeneration does not occur in IL-6 deficient mice unless they receive exogenous IL-6 [20]. We have thus examined the hypothesis that villous atrophy in coeliac disease may represent a disorder of pathological matrix expansion in which IL-6 may be implicated, and have additionally studied expression of other components besides IL-6 involved in T_H_17 pathway responses.

## Methods

### Patients Studied

Formalin-fixed archival jejunal biopsy specimens from the early 1990's were examined, taken from 5 children undergoing diagnostic challenge series (biopsies at diagnosis, on a gluten-free diet and then on gluten challenge). For subsequent studies of extracellular matrix composition and syndecan-1 expression, formalin-fixed duodenal biopsies were obtained from 9 children with active coeliac disease and anti-endomysium and/or anti-tissue transglutaminase antibody positivity. Duodenal biopsies were also obtained from 9 children who had GI symptoms sufficient to warrant endoscopy but were found to have histologically normal tissues and negative coeliac serology (normal controls). Staining patterns for syndecan-1 and sulphated GAGs were then confirmed with a different antibody in a further 12 coeliac biopsies and one control. All children underwent routine diagnostic endoscopy at either the Meyer Hospital, Florence, Italy, the Royal Free Hospital, London, UK or University Hospital Coventry and Warwickshire, Coventry, UK. Patients ranged from 2 to 16 years, with no differences between groups in age distribution.

For studies of IL-6 PCR and immunohistochemistry, snap-frozen biopsies from an additional 9 children with active coeliac disease and 9 histologically normal controls were analysed. For study of Th17 pathway mRNA, frozen biopsies from a further 10 children with coeliac disease and 8 histologically normal controls were obtained. Approval for additional biopsies was obtained from the Research Ethics Committees of the Meyer Hospital and the Royal Free NHS Trust and written informed consent was obtained from parents in all cases. Biopsies from children at University Hospital Coventry were obtained from the University of Birmingham Biomaterials Resource Centre, following approval by their Access Review Panel. Written informed consent for research use had been obtained in all cases from parents prior to the tissues being deposited in the Biomaterials Resource Centre.

Histological assessment of the biopsies from the CD patients showed subtotal or total villous atrophy with increased intraepithelial lymphocytes (IELs), whereas controls showed normal duodenal histology without increase in IELs. No gastrointestinal abnormality was eventually diagnosed in the control patients.

### Tissue Analysis

6 µm sections were cut by cryostat onto poly-L-lysine coated slides and fixed in 4% paraformaldehyde or acetone for IL-6 staining, while GAGs were localised on formalin-fixed specimens. The distribution of sulphated GAGs was studied with a 5 nm gold-conjugated poly-L-lysine probe (1∶100 in phosphate-buffered saline, pH 1.2, British Biocell International, Cardiff, UK) with silver enhancer as previously reported [8],[21]. Serial sections were stained using immunohistochemistry. Endogenous peroxidase activity was blocked with 3% H_2_O_2_ in methanol. Primary mouse monoclonal antibodies used included anti-human HSPG (Seigakaku, UK, dilution 1∶50), Δ-HSPG, recognising short chain HSPG “stubs” after one hour heparitinase digestion (Seikagaku UK, 1∶50), syndecan-1 (Clones MCA681H 1∶100, MCA2459GA 1∶200, Serotec UK). IL-6 was localised using a goat anti-human IL-6 antibody (R&D Systems, UK, 1∶100) followed by rabbit anti-goat immunoglobulins (1∶80, Dako, UK), with staining optimised on sections of tonsil. Bound antigens were localised following manufacturers' instructions using avidin-biotin immunohistochemistry (Vectastain Elite, Vecta). Syndecan-1 clone MCA2459GA was visualised using Novocastra Novolink polymer detection system (Leica Biosystems, UK).

Quantitation of staining intensity for sulphated GAGs, HSPG, Δ-HSPG and syndecan-1 was performed by computerised analysis using ImageJ software (http://rsb.info.nih.gov/ij/) in both epithelial and lamina propria compartments as previously reported [21]. Colour deconvolution was used to separate specific di-amino-benzidine (DAB) or silver staining from counterstain prior to region-of-interest densitometry. Separate quantitation was done in the lamina propria and intraepithelial compartments. For the lamina propria, a single field was drawn around the entire compartment excluding crypts. For the epithelium, high-power images were used, and a mean density was derived for each specimen from quantitation of multiple individual intercellular spaces. Quantitation was on an inverse scale (1–250), based on light transmission, and staining intensity values are presented in reciprocal form (1/transmission intensity ×10^4^ minus 40 (background constant), as previously reported [21]. All statistical analysis was performed on initial transmission intensity data. Quantitation of lamina propria area per unit epithelial length was performed on the same slides using ImageJ quantitation, excluding all epithelial crypts.

Quantitation of positively stained IL-6^+^ cells within the lamina propria was performed per high-powered field (400-fold magnification, equivalent to 0.2 mm^2^), evaluating at least four areas per slide. All sections were blindly and independently examined by two observers, for whom interobserver results varied by less than 8%.

### Rna Isolation For Il-6 Pcr

Biopsies were disrupted using DEPC treated glass tissue grinders and homogenised using QIAshredder spin columns (Qiagen, UK). Total RNA was isolated from biopsies with a monophasic solution of phenol and guanidin isothiocyanate (TRIzol, Invitrogen, CA, USA) and chloroform. Precipitation was obtained with isopropanolol, using RNeasy Micro columns (Qiagen, UK) according to the manufacturer's instructions. RNA concentration was determined by absorption at 260 nm, and the 260/280 nm absorption ratios of the samples were >2. The integrity of the total RNA was assessed using a 1.5% ethidium bromide-stained agarose gel. Only samples with clearly defined 18S and 28S ribosomal RNA peaks were used in the study. To remove genomic DNA contamination, RNA samples were subjected to DNase treatment using a TURBO DNase-free kit (Ambion, UK), following the manufacturer's protocol for extended digestion.

RNA integrity was assessed by subjecting cDNA to amplification by PCR of G3PDH house-keeping gene. Complementary DNA (cDNA) was obtained from 1.5 µg of total RNA using Superscript First-Strand Synthesis System for RT-PCR (Invitrogen, CA, USA) and amplified with Platinum *Taq* DNA Polymerase (Invitrogen, CA, USA), following the manufacturer's instructions. Primers for human IL-6 were provided by R&D Systems, UK. 100 ng of random hexamers, 275 ng of oligo (dT)18 and 50 U of BioScript Reverse Transcriptase were used in each reaction. Duplicate reactions omitting the BioScript Reverse Transcriptase were also carried out. Amplification was performed for 36 cycles, each cycle consisting of denaturation at 94°C, annealing at temperatures appropriate for each primer pairs (55–70°C) and extension at 72°C. CDNA was diluted 1∶4 in TE buffer (10 mM Tris-HCL, 0.1 mM EDTA, pH = 8.0), and stored as aliquots at −20°C. An electrophoresis in ethidium bromide stained 1.2% agarose gel (Agarose I, Amresco, USA) was run and PCR products were visualized by UV. Negative controls (mix without cDNA samples) were run with each PCR assay. The quantitation of PCR products was determined using the Gel Doc 2000 gel documentation system (Bio-Rad, UK). Band density was normalised to the housekeeping gene GAPDH.

### Real-Time Quantitative Pcr (q-Pcr) For Il-17 Pathway Cytokines

Primers (table 1) were designed using Primer3 software (http://frodo.wi.mit.edu/), using stringent maximum self complementary and 3′ self complementary alignment scores to minimise the formation of primer-dimers. Primer sequences for TBP and HRPT1 were down loaded from the public RTPrimerDB^2^ database (http://medgen.UGent.be/rtprimerdb/), RTPrimerDB ID: TBP(2627), HPRT1(984). Primers were synthesised by VH-BIO, UK.

**Table 1:** Primer sequences for real time quantitative PCR studies of IL-17 pathway components and for optimisation of housekeeping genes.

Housekeeping Genes	Entrez gene I.D	Forward Sequence	Reverse Sequence
TBP	6908	TGCACAGGAGCCAAGAGTGAA	CACATCACAGCTCCCCACCA
POLR2A	5430	GATGGGCAAAAGAGTGGACTT	GGGTACTGACTGTTCCCCCT
HPRT1	3251	GCCAGACTTTGTTGGATTTG	CTCTCATCTTAGGCTTTGTATTTTG
GAPDH	2597	AGGTCGGAGTCAACGGATTT	TGGAAGATGGTGATGGGATTT
B2M	567	GGGTTTCATCCATCCGACA	ACACGGCAGGCATACTCATC
ACTB	60	AAACTGGAACGGTGAAGGTG	CCTGTGTGGACTTGGGAGAG
RPLPO	6175	GCAATGTTGCCAGTGTCTG	GCCTTGACCTTTTCAGCAA
PSMB6	5694	AGACTGGGAAAGCCGAGAAG	ATGCGGTCGTGAATAGGTGT
Genes of Interest			
IL17α	3605	AGGAATCACAATCCCACGAA	ACTTTGCCTCCCAGATCACA
IL6	3569	AACCTGAACCTTCCAAAGATGG	TCTGGCTTGTTCCTCACTACT
IL23α	51561	GGACAACAGTCAGTTCTGCTT	CACAGGGCTATCAGGGAGC
TGFβ1	7040	GCGTGCTAATGGTGGAAAC	CGGTGACATCAAAAGATAACCAC

Q-PCR was performed in a Mastercycler ep *realplex* thermal cycler (Eppendorf, UK), using a QuantiTect SYBR Green PCR kit (Qiagen, UK). Amplification reactions were carried out in a volume 20 µl comprising 10 µl of QuantiTect master mix, 0.5 µM of forward and reverse primers in a total volume of 1 µl (with the exception of primers for PSMB6 which were used at a concentration of 0.3 µM), 1 µl cDNA and 7.5 µl water. The PCR parameters comprised an initial hot start step of 15 minutes at 95°C, followed by 45 cycles of 95°C for 30 seconds, 58°C for 30 seconds and 72°C for 30 seconds. PCR product specificity was analysed by melting curve analysis and by running PCR products on 2% ethidium bromide agarose gels. C_t_ values for each gene were acquired using the noise band detection freshold option on the Mastercycler software. Each sample was analyzed in triplicate, and the mean of the C_t_ values was calculated. In addition, RT- samples and no template controls were included in each run to excluded false signals generated by contaminating genomic DNA or primer-dimer formation. To ensure similar PCR amplification efficiencies for each gene, standard curves were constructed using 5 four fold serial dilutions of cDNA. Each gene demonstrated comparable amplification efficiencies of between 90% and 96%.

To determine the most stable reference genes to use in the study, the expression stability of a range of housekeeping genes was analysed using the geNorm VBA applet for Microsoft Excel (http://medgen.ugent.be/∼jvdesomp/genorm/). Housekeeping genes were ranked by pairwise expression stability (M) and the optimum number of reference genes to use for normalisation was determined by using a cut off value of 0.15 for pair wise variation (V). The difference in relative expression between the coeliac and control groups was determined using the ΔC_t_ method. The geometric means of the most stable set of housekeeping genes were used to normalise expression values.

### Statistical Analysis

Statistical analysis for staining intensity was performed using Statgraphics software, comparing group means derived from single mean values for each case. Staining intensity was compared between coeliac disease and normal controls for epithelial and lamina propria compartments using the unmatched *t* test, with level of significance at **p**<0.05. Data are presented as mean ± 95% confidence intervals (95CI). The number of IL-6^+^ cells per high-powered field was expressed as mean ± standard deviation and the data were compared using the unmatched *t* test.

Statistical analyses for real-time PCR were performed using SPSS 13 for Windows. Differences between genotypes were evaluated using a two-tailed Mann-Whitney *U*-test. A *P* value of <0.05 was considered as statistically significant.

## Results

**Figure 1:**
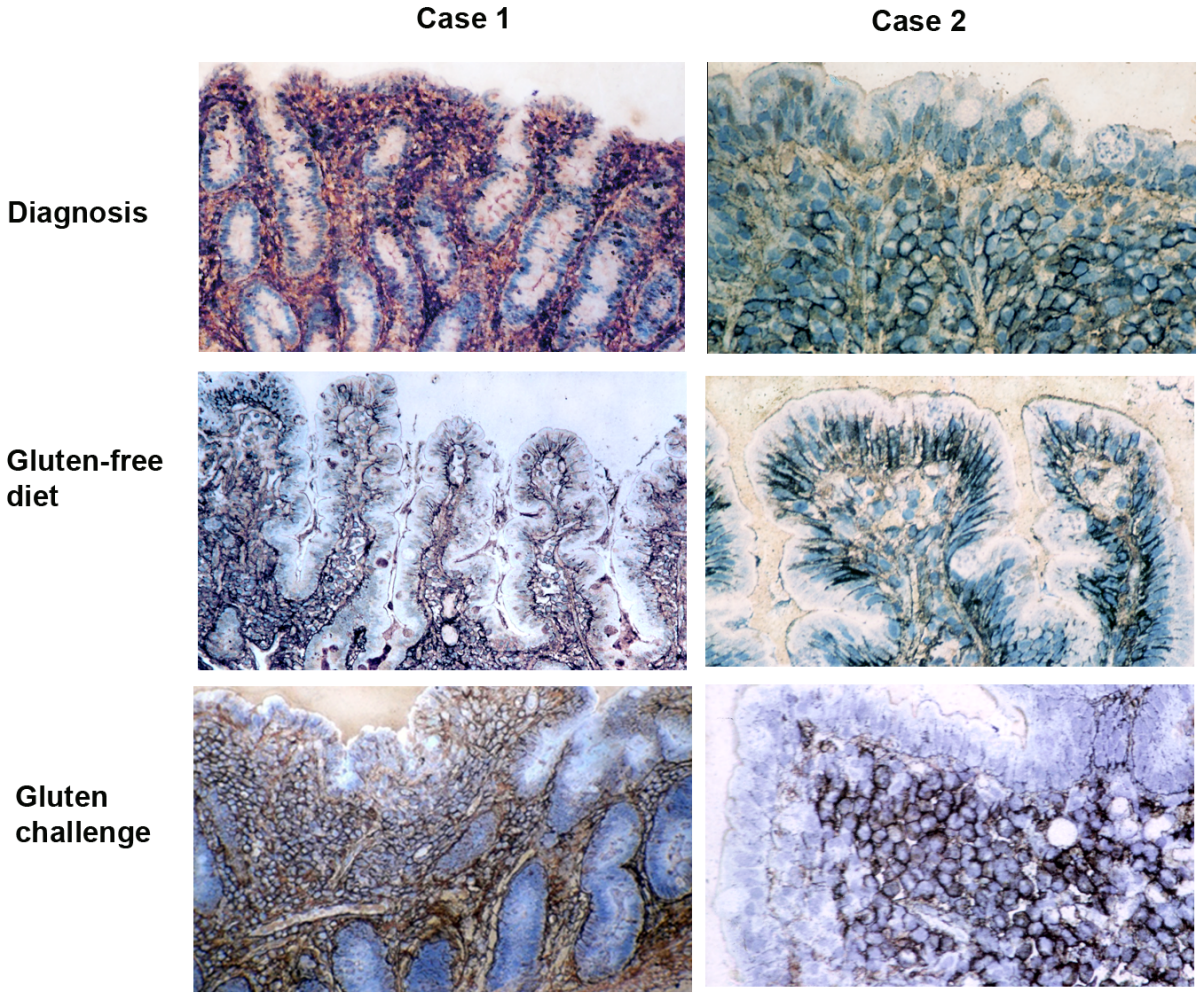
Expression of sulphated GAGs within archival small intestinal biopsies from two patients with coeliac disease during a biopsy challenge series following previous ESPGHAN guidelines. The biopsies shown were taken from the children at initial diagnosis (top row), then on a gluten-free diet (centre row) and finally following gluten challenge (bottom row). The left hand column shows low power views (original magnification ×10) of case 1, and the right hand column high power (x40) views of epithelial staining in case 2. The specimens show decreased epithelial and increased lamina propria sulphated GAG expression in active coeliac disease compared to findings in the same patient on a gluten-free diet. Strong pericellular staining may be seen on aggregated lamina propria mononuclear cells. Similar findings were seen in 3 other cases.

Study of archival specimens from the 5 children undergoing repeated biopsy at diagnosis, on gluten-free diet and on gluten challenge demonstrated similar features in all cases. Epithelial and subepithelial basement membrane sulphated GAG expression was strongly reduced at diagnosis, while expression within the lamina propria appeared increased (Figure 1). All showed contrasting findings on gluten-free diet, similar to those previously reported in normal duodenum [21], with restored GAG expression on the basolateral epithelial surface and in the subepithelial basement membrane, together with restoration of normal architecture. Gluten challenge induced loss of epithelial GAGs and apparent increase in lamina propria expression. One striking feature of the active coeliac lesion was the stronger expression of membrane-bound sulphated GAGs on clustered lamina propria mononuclear cells compared to during gluten-free diet (Figure 1).

### Quantitation Of Mucosal Gag Expression

**Figure 2:**
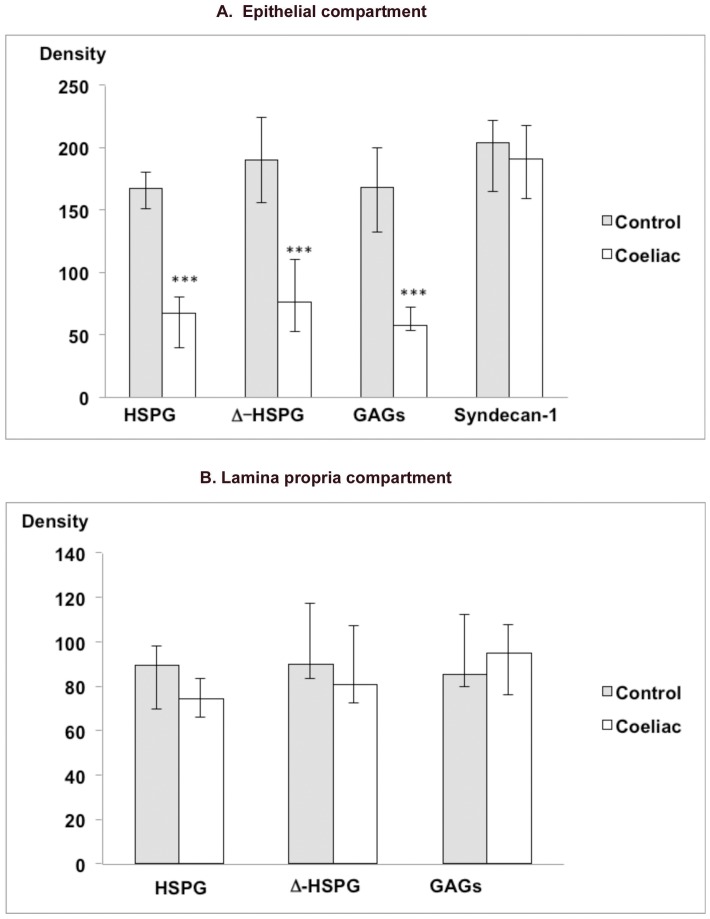
Staining density for glycosaminoglycans within (a) epithelium and (b) lamina propria, in coeliac disease mucosa in comparison to controls. Sulphated GAGs, heparan sulphate proteoglycans (HSPG) and short chain heparan sulphate proteoglycan “stubs” (Δ-HSPG) are shown for both epithelial and lamina propria compartments, while the core protein for epithelial proteoglycans (syndecan-1) is shown for the epithelial compartment alone. Epithelial expression of heparan sulphate and sulphated GAGs is reduced in coeliac disease compared to controls, whereas the core protein syndecan-1 is maintained. Within the lamina propria the density of expression of heparan sulphate and sulphated GAGs is maintained similar to controls. Values shown represent means plus 95% confidence intervals. While the units displayed are derived from reciprocals of initial measurement minus background, statistical comparisons were made on unmodified initial measurements, as previously reported ^21^. Symbols: Significant difference between coeliac and controls: * p<0.05, ** p<0.01, *** p<0.001.

**Figure 3:**
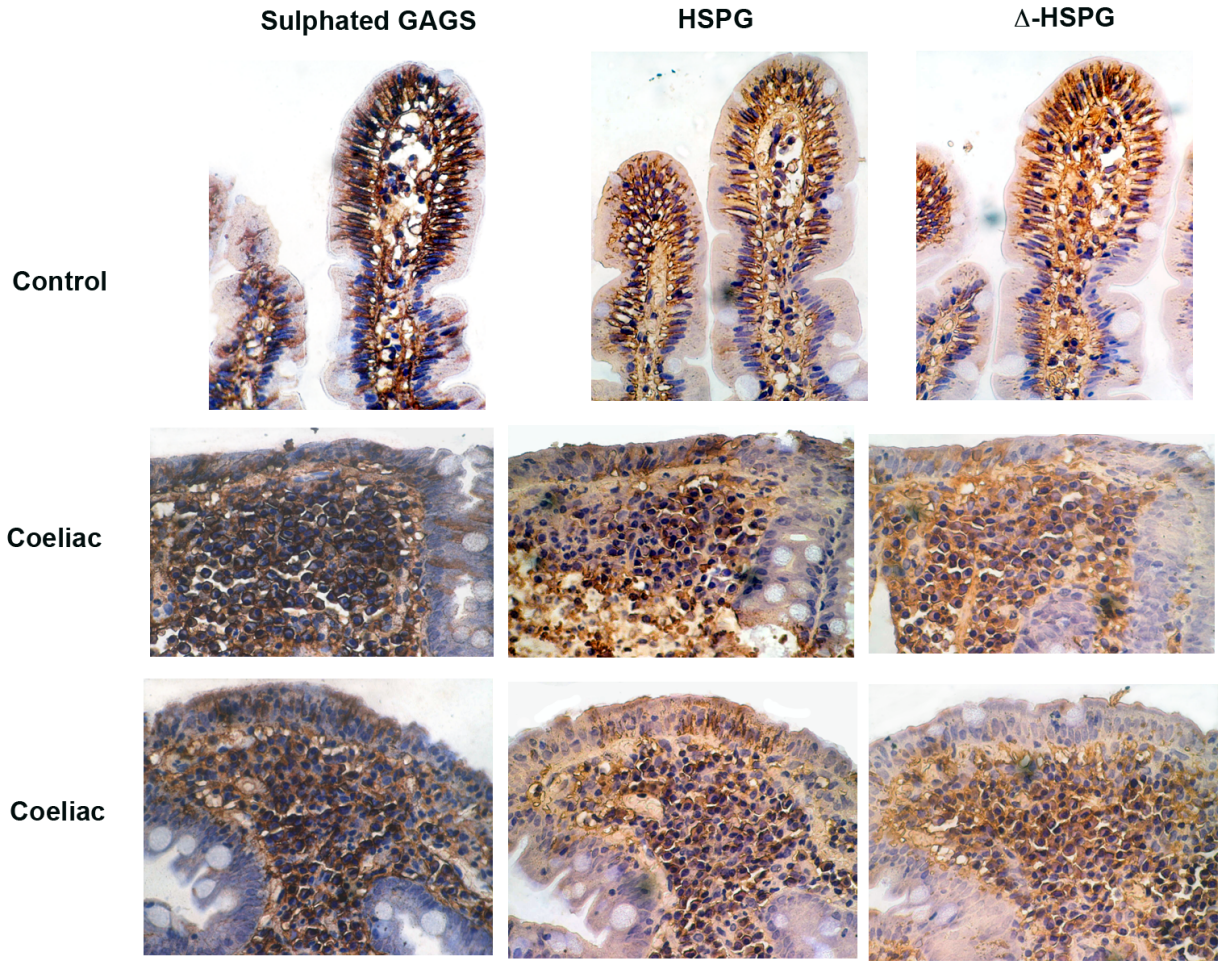
Representative staining for sulphated GAGs, HSPG and Δ-HSPG stubs in control (top row) and coeliac cases (middle and bottom row). All views are at same magnification (original ×40). In the control biopsies, there is strong expression of heparan sulphate and sulphated GAGs within the subepithelial basement membrane and on the basolateral surface of epithelial cells, most marked towards the villous tip. The coeliac biopsies show loss of HSPG and sulphated GAG expression within the basement membrane and from the basolateral epithelial surface. By contrast there is maintained expression within the lamina propria. Additional images from each of the patients studied can be seen in Figures S1 and S2. Quantitative staining intensity data derived from colour deconvoluted images (Figure S3) are shown in Figure 2.

Comparative quantitation of differential GAG/HSPG expression, performed in duodenal biopsies from 9 cases and 9 histologically normal controls, confirmed and extended these findings to demonstrate clear differences between epithelial and lamina propria compartments (Figures 2, 3, Figures S1, S2, S3). Within the epithelium there was significant reduction in GAG chain expression in active coeliac disease, with sulphated GAG, HSPG and short-chain Δ-HSPG staining intensity all decreased compared to controls. By contrast, expression of the core protein molecule syndecan-1 was maintained similar to normal controls and with normal basolateral epithelial localisation. These findings indicate either impairment of epithelial GAG glycan chain extension and sulphation, or their extensive degradation after synthesis.

Contrasting findings within the lamina propria demonstrated evidence of matrix expansion and increased total GAGs. Lamina propria area was substantially increased, consistent with previous reports [2],[3], at 4.1 mm^2^/mm epithelial length (95% CI 3.6–4.6) in comparison to 2.5 mm^2^/mm (2.1–2.9) in controls (**p**<0.0001). Within this compartment, staining intensities for sulphated GAGs, HSPG and Δ-HSPG stubs were maintained overall similar to controls (Figures 2, 3, Figures S1, S2, S3). Thus the overall GAG content of the lamina propria was increased, demonstrating that matrix expansion, at least in part, relates to increased sulphated GAG production.

### Mucosal Expression Of Syndecan-1 (cd138)

**Figure 4:**
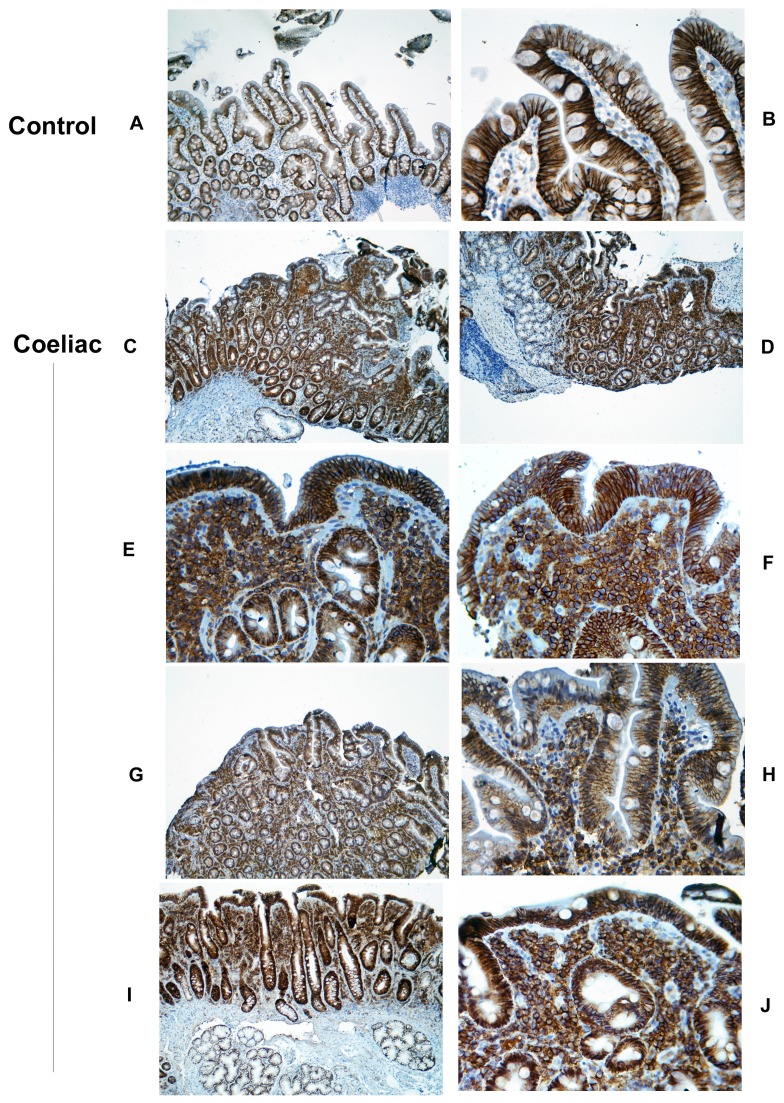
Syndecan-1 (CD138) expression in normal and coeliac mucosa using monoclonal MCA2459GA. **A**. Expression within normal intestine, showing syndecan-1 localises predominantly to the basolateral epithelial surface (original magnification ×10). **B**. Higher power view (x40) of normal duodenum, showing similar epithelial localisation plus the presence of scattered syndecan-1+ plasma cells within the lamina propria. **C,D**. Low power view (x10) of 2 cases of coeliac disease, showing retention of epithelial staining similar to controls, plus additionally a dense confluent expression within the upper lamina propria. **E,F**. Higher power view (x40) of the subepithelial region in these cases. The lamina propria aggregates consist of cells with dense membrane staining (plasma cells), cells with recognisable nuclei and punctate cytoplasmic staining and scattered syndecan-1+ debris (shed ectodomains). **G**–**J**. Findings within the same case in two biopsies showing contrasting areas with containing areas with preserved villous architecture (G,H) and villous atrophy (I,J). G,I are at low power (x10), H,J at high power (x 40). The biopsy with villous atrophy shows dense subepithelial syndecan-1 syncytial expansion in comparison to that with preserved villous architecture. Mucosal syndecan-1 expression from all normal controls and coeliac patients studied is shown in Figure S4.

Identical staining patterns were noted for both MCA681H and MCA2459GA syndecan-1 monoclonals. Syndecan-1 was strongly expressed on the enterocyte basolateral surface in normal small intestine using both antibodies, as previously reported [21]. In contrast to its GAG side chains, syndecan-1 core protein expression was maintained on enterocyte basolateral membrane in coeliac disease, with staining intensity similar to normal small bowel (Figures 2, 4, Figure S4). Localisation of sulphated GAGs on the 12 specimens and one control examined using MCA2459GA showed similar features of epithelial loss and lamina propria preservation as seen in the quantitated specimens (Figure S5).

Within the lamina propria, individual syndecan-1+ plasma cells could be noted within the lamina propria in all histologically normal controls (Figures 4 A,B). Some localised clustering was seen in 2/10 cases. In all 21 coeliac cases, a striking aggregation of syndecan-1+ cells could be seen within the upper lamina propria, forming extensive syncytial sheets which represented the major volume component of the upper lamina propria (Figure 4). At higher magnification, the syncytia comprised both plasma cells with strong epithelial staining, and additional cells with identifiable nuclei and punctate intracellular staining but without strong membrane staining. (Figure 4 E,F,J). In addition, extracellular deposition of syndecan-1 could be seen in all aggregates, representing shed syndecan ectodomains. In cases where serial biopsies showed better preservation of normal villous architecture, there was corresponding reduced syncytial syndecan-1 cell aggregation (Figure 4 G,H compared to I,J).

### Mucosal Expression Of Il-6 And Th17 Pathway Cytokines

**Figure 5:**
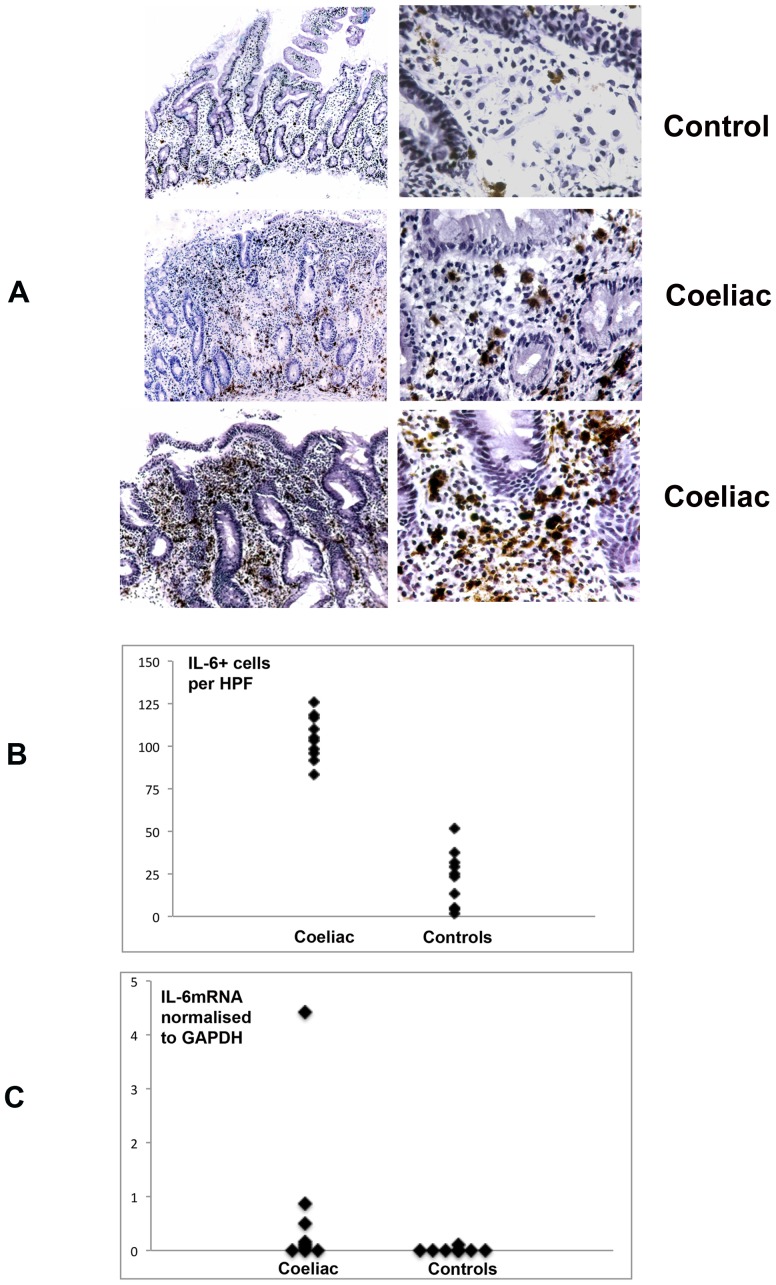
**a**). IL-6 localisation by immunohistochemistry in 2 controls (top panel) and 4 cases of coeliac disease (lower 2 panels). The left column show low power (original magnification ×10) and the right column high power (x40) views. The coeliac patients showed increased numbers of IL-6+ mononuclear cells within the lamina propria but not epithelial compartments. **b**). Quantitative data for mucosal IL-6+ cell density in 9 coeliac and 9 control biopsies, expressed as IL-6+ cells per high power field. **c**). Mucosal IL-6 mRNA expression density normalised to the housekeeping gene GAPDH in matched biopsies from the same cases.

Expression of IL-6 was strongly enhanced within the mucosa of a further 9 children with coeliac disease compared to normal duodenum. Immunohistochemical analysis demonstrated that IL-6 expression was largely confined to cells within the lamina propria (Figure 5a). The IL-6+ cells included small dark staining lymphocyte-like cells and larger irregular cells of characteristic macrophage morphology. The epithelium did not express IL-6 immunoreactivity. The mean density of IL-6^+^ cells within the lamina propria (Figure 5b) was significantly higher in coeliac disease (104.7±13.1/high power field) compared to normal controls (22.2±16.1, **p**<0.05). RT-PCR evaluation in matched biopsies also demonstrated marked increase of IL-6 mRNA in coeliac disease compared to controls (Figure 5c, Figure S6), with mean expression density 508 vs 68/mm^2^, normalised to GAPDH 0.68 vs 0.05 (**p**<0.014).

**Figure 6:**
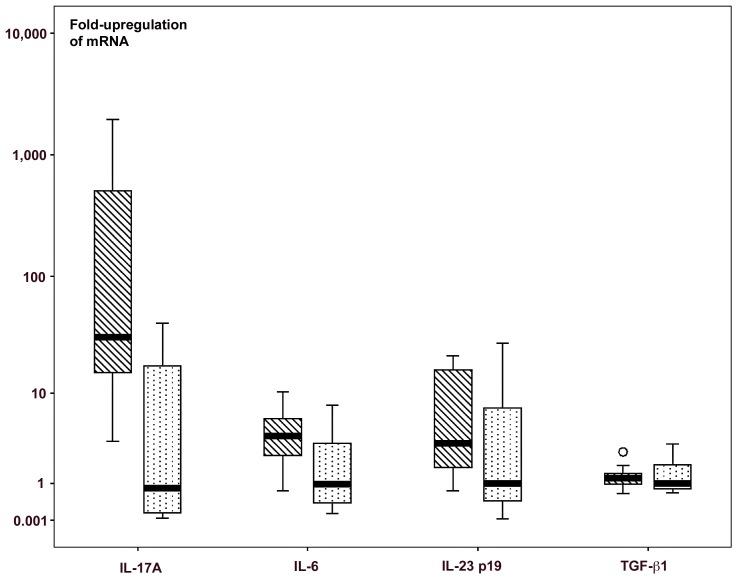
Quantitative analysis of mRNA expression for IL-17α, IL-6, IL-23α and TGF-β1 in biopsies from 10 children with coeliac disease and 8 histologically normal controls. For each cytokine, the left column represents results for coeliac disease and the right column results from controls. Il-17A and IL-6 showed significant increase in coeliac disease. Original data shown in Figure S7.

Quantitative real-time PCR for IL-6, and for other components of the T_H_17 pathway (IL-17, IL-23 p19 and TGF-β1) showed a 30.9-fold median increase for IL-17A (**p**<0.02), a 4.0 -fold increase for IL-6 (**p**<0.05), but without significant increase for IL-23 p19 and TGF-β1 mRNA (Figure 6, Figure S7).

## Conclusions

Matrix changes in coeliac disease are distinct from in IBD, which shows widespread loss of mucosal sulphated GAGs [22]. In coeliac disease, GAG disruption is limited to the epithelial compartment, while the lamina propria shows undiminished sulphated GAG expression. By contrast, unsulphated hyaluronan has been shown to be patchily decreased in coeliac mucosa [23]. Limitations imposed by biopsy size currently preclude more formal glycobiochemical assessment. While we measured tissue area as a proxy for volume, this cannot be directly determined from biopsy sections, though previous mathematical modelling suggests an approximate doubling of volume in coeliac disease [2].

Loss of epithelial HSPG promotes protein leak into the lumen [7],[8]. Our findings of lost epithelial HSPG will contribute to the increased albumin leak in coeliac disease [24]. The rarity of coeliac crisis, characterised by severe PLE, is likely due to retardation of albumin within the coeliac lamina propria in an expanded anionic gel [24], comprising aggregates of negatively charged syndecan-expressing leukocytes in a matrix of sulphated GAGs. Acute infection, the usual trigger of coeliac crisis, will induce vascular albumin leak and upregulation of mucosal matrix degrading metalloproteases, thus delivering unrestricted albumin to the HSPG-depleted basement membrane and epithelium to promote PLE.

We found that much of the mucosal GAGs in coeliac disease localises within syncytial aggregates of syndecan-1+ plasma cells, cell remnants with intracytoplasmic staining and shed syndecan ectodomains. Classic studies of gluten-induced changes in coeliac disease showed that villous atrophy is caused by swelling of the intervillous ridges [3] and that gluten-induced matrix deposition in the lamina propria was characterised by dense aggregation of plasma cells - many lysed with loss of their limiting membrane [4]. These features are entirely consistent with our findings.

We hypothesise that our findings, of syndecan-1+ cell syncytial aggregation with excess GAG production, recapitulate core elements of the invertebrate encapsulation reaction, in which insect haemocytes form palisades around an invading pathogen or object too large to phagocytose [13]–[15],[25],[26]. Many initially binding cells undergo lysis, while subsequent adhering cells form extensive sheets to wall off the pathogen. GAG production is stimulated [13]–[15],[26], providing a physiological barrier to nutrient absorption. Recruited cells are characterised by syndecan expression [27], required for invertebrate haemocyte binding to laminin and cell cluster formation [28]. Our findings in coeliac mucosa show a similar palisading of syndecan-expressing leukocytes to form syncytial aggregates, together with a lysis of a proportion of these cells, with loss of their plasma membrane. Sulphated GAG including HSPG accumulated around these aggregates in an expansion of the lamina propria. In insects, the GAG production and matrix expansion is limited by a subsequent melanisation reaction dependent on the enzyme prophenoloxidase [29], in which haemocyte responses are downregulated by α-melanocyte stimulating hormone (α-MSH) [30]. In coeliac mucosa the gliadin-induced IL-6 response is similarly attenuated by α-MSH [31].

A contrasting interpretation of our findings is that the plasma cell aggregates may represent a peripheral niche for plasma cell survival [32]. Membrane syndecan-1 is critical for long-term survival of plasma cells, binding to mediators such as APRIL and CXCL12 that prevent apoptosis [33]. IL-6 also plays an important role in maintaining plasma cell survival in the periphery [32]. While such a niche cannot be excluded, it is notable that many of the plasma cells show evidence of lysis [3], a characteristic of invertebrate encapsulation [13]–[15].

The encapsulation reaction has additional specific similarities to coeliac disease, as encapsulation is dependent on both insect transglutaminase expression [34] and two gliadin-like early encapsulation proteins [35]. Could exogenous gliadin thus trigger this response? It is notable in this context that the glutenin moiety of wheats provides an effective barrier to predation by most insect species, and there are notably few successful phytophagous predators. Insects able to predate wheat share a common ability to predigest gluten with salivary enzymes, which has evolved separately in species from the northern and southern hemispheres. The Sunn bug *Eurygaster integricepts*, a major pest in southern Europe, north Africa and Asia, secretes glutenin-specific serine proteases in its saliva, cleaving gliadin at glutamine residues between consensus hexapeptide and nonapeptide repeats [36],[37]. The New Zealand wheat bug, *Nysius huttoni*, secretes a more specific salivary proteinase species, cleaving between glutamine and glycine residues within hexapetide repeats [38]. Insect-predated wheat shows abnormalities in the ability to make dough, because of this molecular disruption [36]–[38]. The role of glutenins in dough production was critical in Neolithic times, leading to early selection for high glutenin wheats [39]. It would be ironic if the dietary constituent that played such a role in advancing human socio-economic development had evolved in plant host defense as a trigger of innate immunity in predators.

Our finding of shed syndecan ectodomains may provide a link between innate immune response to gliadins and the MHC-restricted adaptive immune response characteristic of coeliac disease. One unexplained mechanism in coeliac disease is the triggering of extracellular release of TG2 and its subsequent activation, necessary for deamidation of gluten and adaptive immune responses [40]. Syndecan shedding is critical in the release of TG2 from cells into the extracellular milieu, through specific binding of TG2 to syndecan HS chains [41]. In enterocytes the uptake of α(2)-gliadin-mer is facilitated by the small GTP-ase Rab5 [42], which interacts with the cytoplasmic tail of syndecan-1 to regulate ectodomain shedding [43]. Following TG2 release, the presence of calcium and inhibition of local oxidation are critical for its activation [40]. Known functions of HS include local sequestration of cations and binding of superoxide dismutase [6], thus potentially allowing shed syndecan ectodomains to provide a milieu favourable to TG2 activation.

Excess mucosal IL-6 production has been shown to occur in coeliac disease and to differentiate it from non-coeliac gluten intolerance, in which enhanced innate immune responses also occur but without adaptive changes [16]. We did not formally characterise the IL-6 producing cells, but noted lymphocyte-like and macrophage-like populations, without evidence of epithelial immunoreactivity. IL-6 has multiple functions in immune regulation and inflammation, and is particularly important in autoimmune diseases. It regulates the balance between generation of IL-17 producing cells and T regulatory cells towards an inflammatory response, by promoting development of Th17 cells in concert with TGF-β while blocking TGF-β induced production of Treg cells [44]. Other Th17 pathway components are increased within coeliac mucosa [45],[46], with exposure to IL-15 promoting a Th17 response to gliadin by monocytes [47]. Whether other Th17 cytokines may contribute to pathological matrix expansion, as does IL-6 [17]–[20], is currently unclear.

Gliadin peptides have been shown induce a significant innate immune response in epithelium, mucosal macrophages and dendritic cells in humans, both with and without coeliac disease, as well as in murine cells [45],[48]–[55]. We propose that elements of this innate response contribute to the characteristic architectural disturbance in coeliac disease in a fashion analogous to the insect encapsulation reaction, through cell recruitment and induced matrix expansion. In refractory coeliac disease, where villous atrophy persists despite gluten free diet, mucosal IL-6 expression is further increased [56] and its systemic release enhanced, especially in enteropathy associated T cell lymphoma [57]. IL-6 may represent a pivotal target for immunotherapy in refractory coeliac disease.

## Supporting Information

Figure S1 Serial sections from the 9 control duodenal biopsies studied for matrix quantitation, stained for heparan sulphate proteoglycan (HSPG), short-chain heparan sulphate (Δ-HSPG) by immunohistochemistry and sulphated GAGs by specific charge-based immunohistochemistry (original magnification ×20). (TIF)

Figure S2 Serial sections from the 9 coeliac duodenal biopsies studied for matrix quantitation, stained for heparan sulphate proteoglycan (HSPG), short-chain heparan sulphate (Δ-HSPG) by immunohistochemistry and sulphated GAG by specific charge-based immunohistochemistry (original magnification ×20). (TIF)

Figure S3 Colour-deconjugated images, separated by ImageJ, of the 9 control and 9 coeliac specimens used for densitometric assessment. Deconvoluted DAB staining is shown for HSPG (upper panel) and Δ-HSPG (middle panel), while deconvoluted silver staining is shown for sulphated GAGs (lower panel). The images show preservation of basement membrane and basolateral epithelial expression in controls compared to loss in coeliac disease. (TIF)

Figure S4 Staining for syndecan-1 (CD138) with two monoclonals. The upper 3 rows show syndecan-1 staining for staining in normal controls using clone MCA681H. The same monoclonal is shown for the matched coeliac disease biopsies in rows 3–6 (all at original magnification ×20). These specimens were used for the matrix quantitation studies shown in Figure 2. The slides demonstrate preserved epithelial basolateral staining in the coeliac biopsies together with the presence of confluent subepithelial aggregates of CD138+ plasma cells. Rows 7–10 demonstrate staining for syndecan-1 using clone MCA2459GA in 12 additional duodenal biopsies from children with coeliac disease obtained from the University of Birmingham Biomaterials Resource Centre (original magnification ×20). The staining was visualised with the Novocastra Novolink polymer detection system, giving a darker reaction product. The single panel in row 11 is an additional control specimen stained with clone MCA2459GA. (TIF)

Figure S5 Staining for sulphated GAGs in the 12 coeliac biopsies obtained from the University of Birmingham Biomaterials Resource Centre, showing similar features of maintained lamina propria but lost epithelial staining to those seen in the quantitation study specimens (Figure S2). (TIF)

Figure S6 RT-PCR analysis of IL-6 mRNA in normal controls (upper panel) and coeliac disease (lower panel). Quantitative data is shown in Figure 5c. (TIF)

Figure S7 A. Determination of the most stable housekeeping gene for IL-17 pathway PCR studies. The tata binding protein, proteosomal membrane bound protein 6 and β2-microglobulin genes performed most stably and were used as references. B. Use of the stable housekeeping gene panel to determine relative expression of IL-17 pathway genes. C. Determination of fold-difference expression for IL-17 pathway genes. These data were used to formulate Figure 6. (TIF)

## References

[1] Abadie V, Sollid LM, Barreiro LB, Jabri B (2011) Integration of genetic and immunological insights into a model of celiac disease pathogenesis. Annu Rev Immunol 29: 493–525. 21219178 10.1146/annurev-immunol-040210-092915

[2] Dhesi I, Marsh MN, Kelly C, Crowe P (1984) Morphometric analysis of small intestinal mucosa II. Determination of lamina propria volumes, plasma cell and neutrophil populations within control and coeliac disease mucosa. Virchow's Arch 403: 173–80. 10.1007/BF00695233 6426161

[3] Loehry CA, Creamer B (1969) Three-dimensional structure of the human small intestinal mucosa in health and disease. Gut 10: 6–12. 5784163 10.1136/gut.10.1.6 PMC1552697

[4] Shiner M (1973) Ultrastructural changes suggestive of immune reactions in the jejunal mucosa of coeliac children following gluten challenge. Gut 14: 1–12. 4692249 10.1136/gut.14.1.1 PMC1412582

[5] Comper WD, Laurent TC (1978) Physiological function of connective tissue polysaccharides. Physiol Rev 58: 255–315. 414242 10.1152/physrev.1978.58.1.255

[6] Bishop JR, Schuksz M, Esko JD (2007) Heparan sulfate proteoglycans fine-tune mammalian physiology. Nature 446: 1030–1037. 17460664 10.1038/nature05817

[7] Bode L, Murch S, Freeze HH (2006) Heparan sulfate plays a central role in a dynamic in vitro model of protein-losing enteropathy. J Biol Chem 281: 7809–7815. 16434407 10.1074/jbc.M510722200

[8] Bode L, Salvestrini C, Park PW, Li JP, Esko JD, et al (2008) Heparan sulfate and syndecan-1 are essential in maintaining intestinal epithelial barrier function. J Clin Invest 118: 229–238. 18064305 10.1172/JCI32335 PMC2117765

[9] Bahn RS, Heufelder AE (1993) Pathogenesis of Graves' ophthalmopathy. N Engl J Med 329: 1468–1475. 8413459 10.1056/NEJM199311113292007

[10] Antunes SL, Gallo ME, de Almeida SM, Mota EE, Pelajo M, et al (1999) Dermal extracellular matrix in cutaneous leprosy lesions. Int J Lepr Other Mycobact Dis 67: 24–35. 10407626

[11] Gressner AM (1994) Activation of proteoglycan synthesis in injured liver—a brief review of molecular and cellular aspects. Eur J Clin Chem Clin Biochem 32: 225–237. 8038263

[12] Huang J, Olivenstein R, Taha R, Hamid Q, Ludwig M (1999) Enhanced proteoglycan deposition in the airway wall of atopic asthmatics. Am J Respir Crit Care Med 160: 725–729. 10430752 10.1164/ajrccm.160.2.9809040

[13] Salt G (1963) The defence reactions of insects to metazoan parasites. Parasitology 53: 527–642. 14080003 10.1017/s0031182000073960

[14] Williams MJ (2007) Drosophila hemopoiesis and cellular immunity. J Immunol 178: 4711–4716. 17404248 10.4049/jimmunol.178.8.4711

[15] Vinson SB (1990) How parasitoids deal with the immune system of their host: an overview. Arch Insect Biochem Physiol 13: 3–27.

[16] Sapone A, Lammers KM, Casolaro V, Cammarota M, Giuliano MT, et al (2011) Divergence of gut permeability and mucosal immune gene expression in two gluten-associated conditions: celiac disease and gluten sensitivity. BMC Med 9: 23. 21392369 10.1186/1741-7015-9-23 PMC3065425

[17] Duncan MR, Berman B (1991) Stimulation of collagen and glycosaminoglycan production in cultured human adult dermal fibroblasts by recombinant human interleukin 6. J Invest Dermatol 97: 686–692. 1940439 10.1111/1523-1747.ep12483971

[18] Chen B, Tsui S, Smith TJ (2005) IL-1β induces IL-6 expression in human orbital fibroblasts: identification of an anatomic-site specific phenotypic attribute relevant to thyroid-associated ophthalmopathy. J Immunol 175: 1310–1319. 16002736 10.4049/jimmunol.175.2.1310

[19] Morton RS, Dongari-Bagtzoglou AI (1999) Regulation of gingival fibroblast interleukin-6 secretion by cyclosporine A. J Periodontol 70: 1464–1471. 10632522 10.1902/jop.1999.70.12.1464

[20] Cressman DE, Greenbaum LE, DeAngelis RA, Ciliberto C, Furth EE, et al (1996) Liver failure and defective hepatocyte regeneration in interleukin-6-deficient mice. Science 274: 1379–1383. 8910279 10.1126/science.274.5291.1379

[21] Amadi B, Fagbemi AO, Kelly P, Mwiya M, Torrente F, et al (2009) Reduced production of sulfated glycosaminoglyans occurs in Zambian children with kwashiorkor but not marasmus. Am J Clin Nutr 89: 592–600. 19116330 10.3945/ajcn.2008.27092

[22] Murch SH, MacDonald TT, Walker-Smith JA, Levin M, Lionetti P, et al (1993) Disruption of sulphated glycosaminoglycans in intestinal inflammation. Lancet 341: 711–4. 8095623 10.1016/0140-6736(93)90485-y

[23] Kemppainen T, Tammi R, Tammi M, Agren U, Julkunen R, et al (2005) Elevated duodenal expression of hyaluronan and its CD44 receptor in the duodenal mucosa of coeliac patients. Histopathology 2005 46: 64–72. 10.1111/j.1365-2559.2005.02001.x 15656888

[24] Lavö B, Knutson L, Lööf L, Odlind B, Hällgren R (1990) Signs of increased leakage over the jejunal mucosa during gliadin challenge of patients with coeliac disease. Gut 31: 153–157. 2179068 10.1136/gut.31.2.153 PMC1378371

[25] Parrinello N, Patricolo E (1984) Inflammatory-like reaction in the tunic of *Ciona intestinalis* (Tunicata). II. Capsule components. Biol Bull 167: 238–250.

[26] Crompton DW (1964) The envelope surrounding *Polymorphus minutus* (Goeze, 1782) (Acanthocephala) during its development in the intermediate host, *Gammarus pulex* . Parasitology 54: 721–735. 14227633 10.1017/s0031182000082731

[27] Spring J, Paine-Saunders S, Hynes RO, Bernfield M (1994) *Drosophila* syndecan: conservation of a cell-surface heparan sulphate proteoglycan. Proc Natl Acad Sci USA 91: 3334–3338. 8159748 10.1073/pnas.91.8.3334 PMC43571

[28] Narita R, Yamashita H, Goto A, Imai H, Ichihara S, et al (2004) Syndecan-dependent binding of *Drosophila* hemocytes to laminin α3/5 chain LG4-5 modules: potential role in sessile hemocyte islets formation. FEBS Letters 576: 127–132. 15474023 10.1016/j.febslet.2004.08.073

[29] Chen CC, Laurence BR (1987) In vitro study on humoral encapsulation of microfilariae: effects of diethyldithiocarbamate and dopachrome on the reaction. Int J Parasitol 17: 789–794. 3032833 10.1016/0020-7519(87)90060-9

[30] Grimaldi A, Tettamanti G, Congiu T, Girardello R, Malagoli D, et al (2012) The main actors involved in parasitization of *Heliothis virescens* larva. Cell Tissue Res 350: 491–502. 23053052 10.1007/s00441-012-1503-8

[31] Colombo G, Buffa R, Bardella MT, Garofalo L, Carlin A, et al (2002-3) Anti-inflammatory effects of α-melanocyte-stimulating hormone in celiac intestinal mucosa. Neuroimmunomodulation 10: 208–216. 12584408 10.1159/000068323

[32] Radbruch A, Muehlinghaus G, Luger EO, Inamine A, Smith KG, et al (2006) Competence and competition: the challenge of becoming a long-lived plasma cell. Nat Rev Immunol 6: 741–750. 16977339 10.1038/nri1886

[33] Reijmers RM, Spaargaren M, Pals ST (2013) Heparan sulfate proteoglycans in the control of B cell development and the pathogenesis of multiple myeloma. FEBS J 280: 2180–2193. 23419151 10.1111/febs.12180

[34] Wang Z, Wilhelmsson C, Hyrsl P, Loof TG, Dobes P, et al (2010) Pathogen entrapment by transglutaminase – a conserved early innate immune mechanism. PLOS Pathogens 6 (2): e1000763. 10.1371/journal.ppat.1000763 PMC2820530 20169185

[35] Cho MY, Lee HS, Lee KM, Homma K, Natori S, et al (1999) Molecular cloning and functional properties of two early-stage encapsulation-relating proteins from the coleopteran insect, *Tenebrio molitor* larvae. Eur J Biochem 262: 737–744. 10411635 10.1046/j.1432-1327.1999.00416.x

[36] Konarev AV, Beaudoin F, Marsh J, Vilkova NA, Nefedova LI, et al (2011) Characterization of a glutenin-specific serine proteinase of Sunn bug *Eurygaster integricepts* Put. J Agric Food Chem 59: 2462–70. 21323348 10.1021/jf103867g

[37] Hosseininaveh V, Bandani A, Hosseininaveh F (2009) Digestive proteolytic activity in the Sunn pest, *Eurygaster integriceps* . J Insect Sci 9: 1–11. 10.1673/031.009.7001 PMC3011966 20053125

[38] Every D, Sutton KH, Shewry PR, Tatham AS, Coolbear T (2005) Specificity of action of an insect proteinase purified from wheat grain infested by the New Zealand wheat bug, *Nysius huttoni* . J Cereal Sci 42: 185–191.

[39] Greco L (1997) From the neolithic revolution to gluten intolerance: benefits and problems associated with the cultivation of wheat. J Pediatr Gastroenterol Nutr 24: S14–16. 9161969 10.1097/00005176-199700001-00005

[40] Qiao SW, Iversen R, Ráki M, Sollid LM (2012) The adaptive immune response in celiac disease. Semin Immunopathol 34: 523–540. 22535446 10.1007/s00281-012-0314-z

[41] Wang Z, Collighan RJ, Pytel K, Rathbone DL, Li X, et al (2012) Characterization of heparin-binding site of tissue transglutaminase: its importance in cell surface targeting, matrix deposition, and cell signaling. J Biol Chem 287: 13063–13083. 22298777 10.1074/jbc.M111.294819 PMC3339925

[42] Hayashida K, Stahl PD, Park PW (2008) Syndecan-1 ectodomain shedding is regulated by the small GTPase Rab5. J Biol Chem 283: 35435–35444. 18957427 10.1074/jbc.M804172200 PMC2602919

[43] Schumann M, Richter JF, Wedell I, Moos V, Zimmermann-Kordmann M, et al (2008) Mechanisms of epithelial translocation of the α(2)-gliadin-33mer in coeliac sprue. Gut 57: 747–754. 18305066 10.1136/gut.2007.136366

[44] Kimura A, Kishimoto T (2010) IL-6: regulator of Treg/Th17 balance. Eur J Immunol 40: 1830–1835. 20583029 10.1002/eji.201040391

[45] Sapone A, Lammers KM, Mazzarella G, Mikhailenko I, Cartenì M, et al (2009) Differential mucosal IL-17 expression in two gliadin-induced disorders: gluten sensitivity and the autoimmune enteropathy celiac disease. Int Arch Allergy Immunol 152: 75–80. 19940509 10.1159/000260087 PMC2956008

[46] Fernández S, Molina IJ, Romero P, González R, Peña J, et al (2011) Characterization of gliadin-specific Th17 cells from the mucosa of celiac disease patients. Am J Gastroenterol 106: 528–538. 21206487 10.1038/ajg.2010.465

[47] Harris KM, Fasano A, Mann DL (2010) Monocytes differentiated with IL-15 support Th17 and Th1 responses to wheat gliadin: implications for celiac disease. Clin Immunol 135: 430–439. 20153259 10.1016/j.clim.2010.01.003 PMC2868103

[48] Bernardo D, Garrote JA, Fernández-Salazar L, Riestra S, Arranz E (2007) Is gliadin really safe for non-coeliac individuals? Production of interleukin 15 in biopsy culture from non-coeliac individuals challenged with gliadin peptides. Gut 56: 889–890. 17519496 10.1136/gut.2006.118265 PMC1954879

[49] Nanayakkara M, Lania G, Maglio M, Discepolo V, Sarno M, et al (2013) An undigested gliadin peptide activates innate immunity and proliferative signaling in enterocytes: the role in celiac disease. Am J Clin Nutr 98: 1123–1135. 23966426 10.3945/ajcn.112.054544

[50] Tucková L, Novotná J, Novák P, Flegelová Z, Kveton T, et al (2002) Activation of macrophages by gliadin fragments: isolation and characterization of active peptide. J Leukoc Biol 71: 625–631. 11927649

[51] Jelínková L, Tucková L, Cinová J, Flegelová Z, Tlaskalová-Hogenová H (2004) Gliadin stimulates human monocytes to production of IL-8 and TNF-α through a mechanism involving NF-κB. FEBS Lett 571: 81–85. 15280021 10.1016/j.febslet.2004.06.057

[52] Londei M, Ciacci C, Ricciardelli I, Vacca L, Quaratino S, et al (2005) Gliadin as a stimulator of innate responses in celiac disease. Mol Immunol 42: 913–918. 15829281 10.1016/j.molimm.2004.12.005

[53] Rakhimova M, Esslinger B, Schulze-Krebs A, Hahn EG, Schuppan D, et al (2009) In vitro differentiation of human monocytes into dendritic cells by peptic-tryptic digest of gliadin is independent of genetic predisposition and the presence of celiac disease. J Clin Immunol 2009 29: 29–37. 10.1007/s10875-008-9228-x 18696220

[54] Chladkova B, Kamanova J, Palova-Jelinkova L, Cinova J, Sebo P, et al (2011) Gliadin fragments promote migration of dendritic cells. J Cell Mol Med 15: 938–948. 20406323 10.1111/j.1582-4934.2010.01066.x PMC3922678

[55] Palová-Jelínková L, Dáňová K, Drašarová H, Dvořák M, Funda DP, et al (2013) Pepsin digest of wheat gliadin fraction increases production of IL-1β via TLR4/MyD88/TRIF/MAPK/NF-κB signaling pathway and an NLRP3 inflammasome activation. PLoS One 8: e62426. 23658628 10.1371/journal.pone.0062426 PMC3639175

[56] Caruso R, Marafini I, Sedda S, Del Vecchio Blanco G, Giuffrida P, et al (2014) Analysis of the cytokine profile in the duodenal mucosa of refractory coeliac disease patients. Clin Sci (Lond) 126: 451–458. 24125165 10.1042/CS20130478

[57] Tack GJ, van Wanrooij RL, Von Blomberg BM, Amini H, Coupe VM, et al (2012) Serum parameters in the spectrum of coeliac disease: beyond standard antibody testing—a cohort study. BMC Gastroenterol 12: 159. 23145841 10.1186/1471-230X-12-159 PMC3579729

