# Supplementary figures and images for: Matrix Expansion and Syncytial Aggregation of Syndecan-1+ Cells Underpin Villous Atrophy in Coeliac Disease
Source: PLoS One. 2014 Sep 8;9(9):e106005. doi: 10.1371/journal.pone.0106005 (PMC4157760; doi:10.1371/journal.pone.0106005)

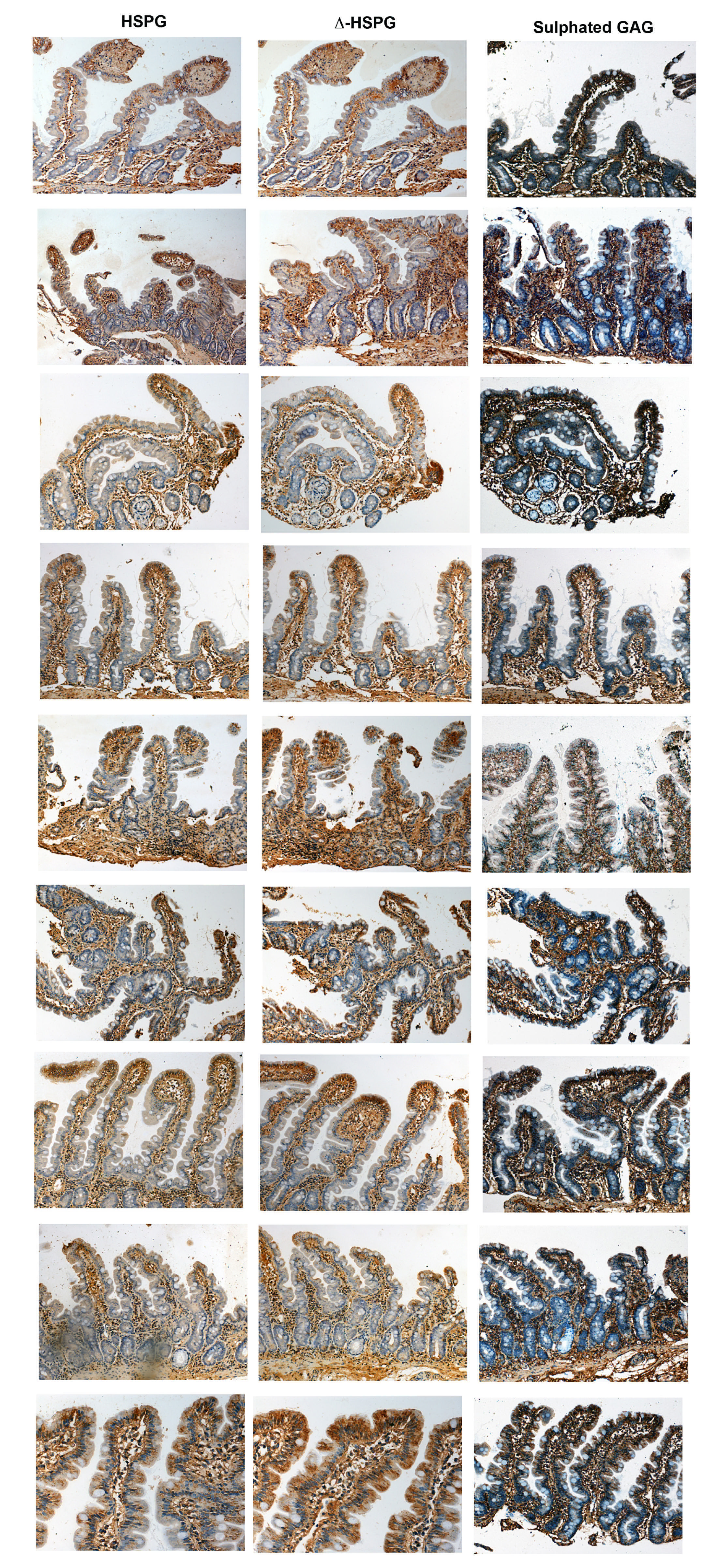

Supplement: Figure S1 — (TIF) [file pone.0106005.s001.tif]

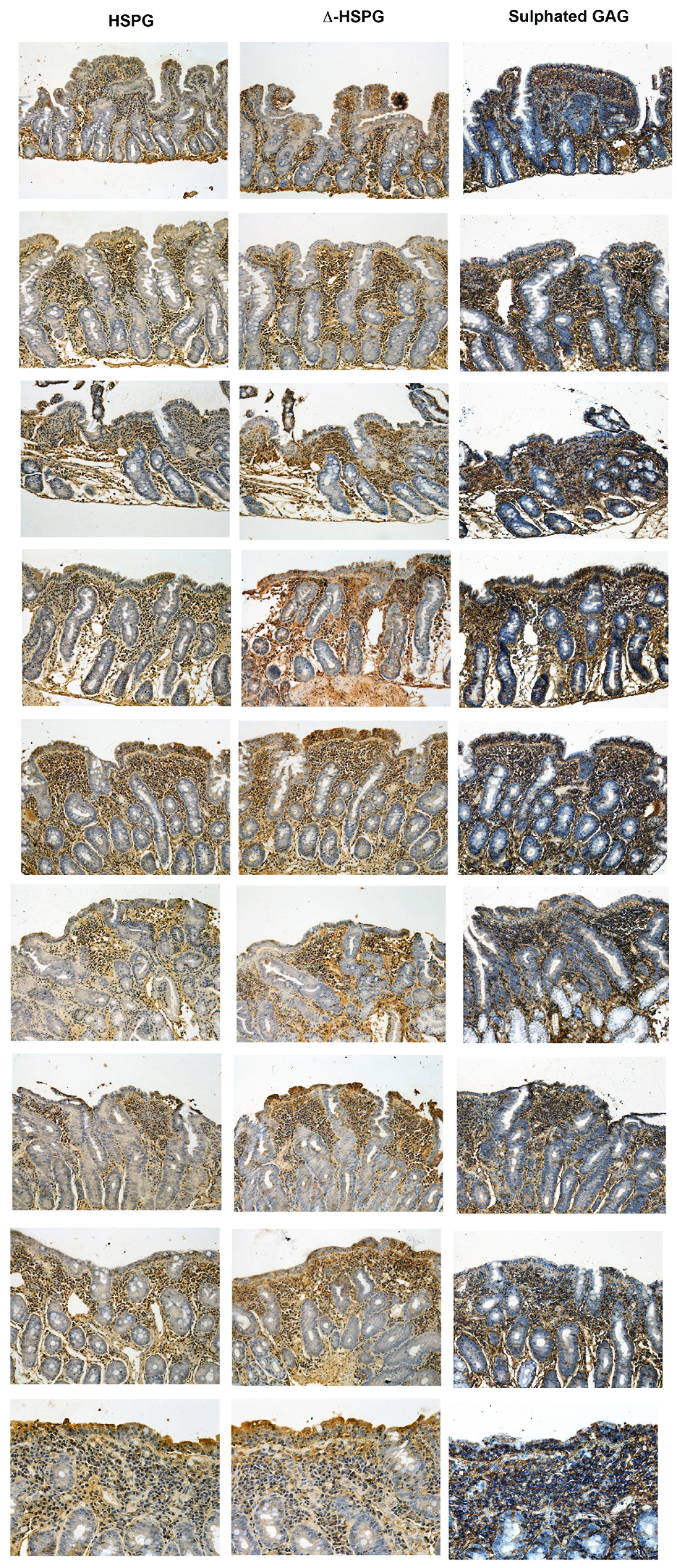

Supplement: Figure S2 — (TIF) [file pone.0106005.s002.tif]

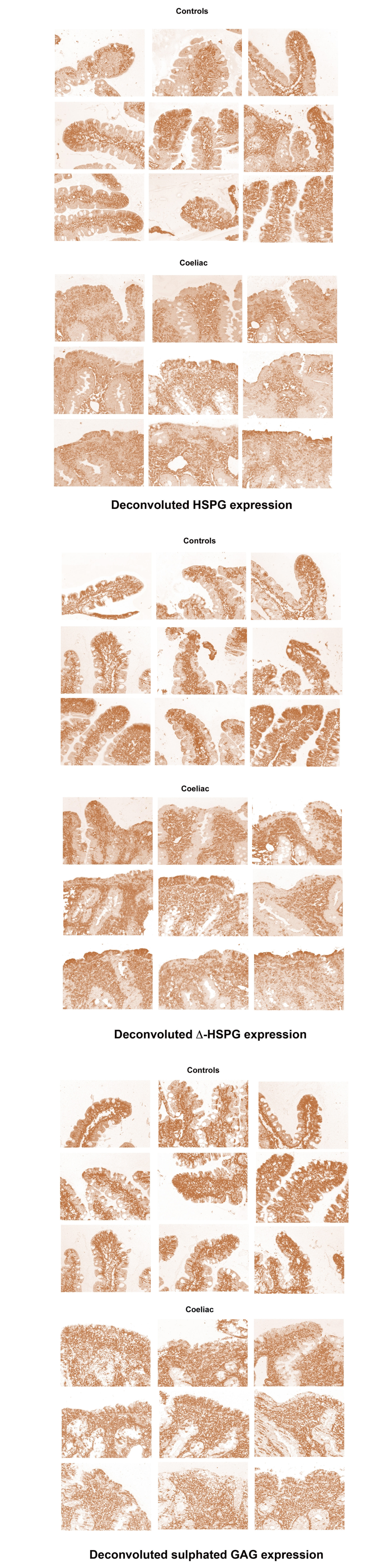

Supplement: Figure S3 — (TIF) [file pone.0106005.s003.tif]

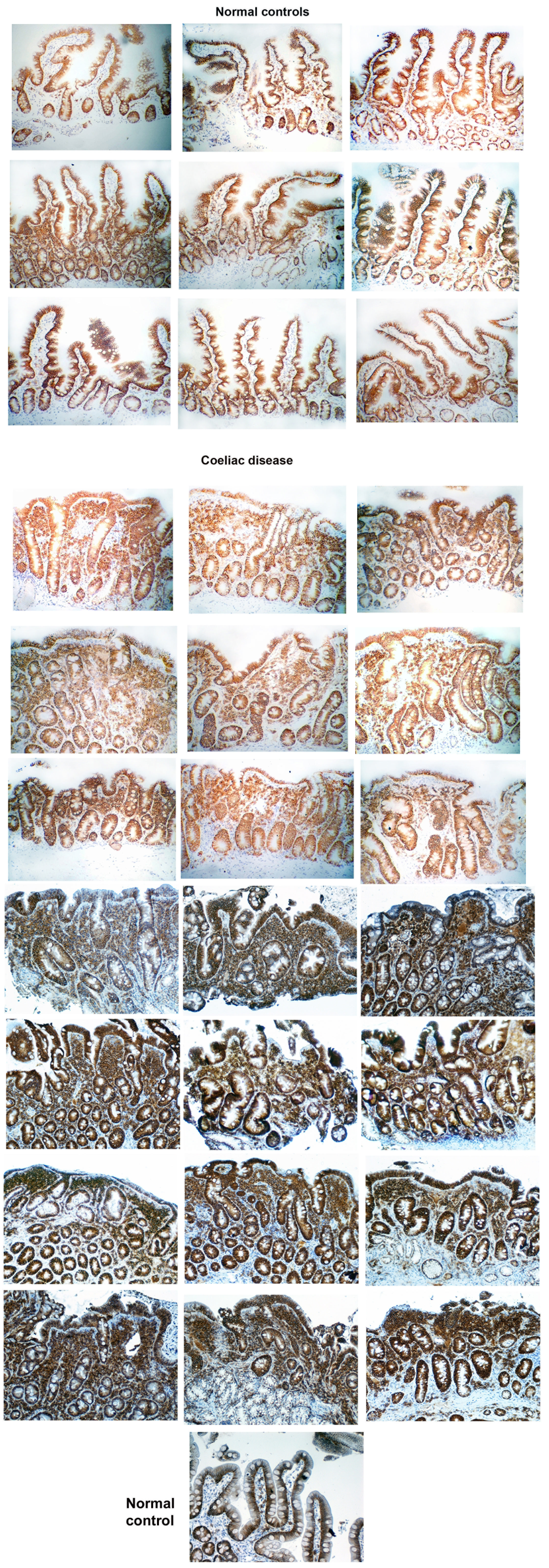

Supplement: Figure S4 — (TIF) [file pone.0106005.s004.tif]

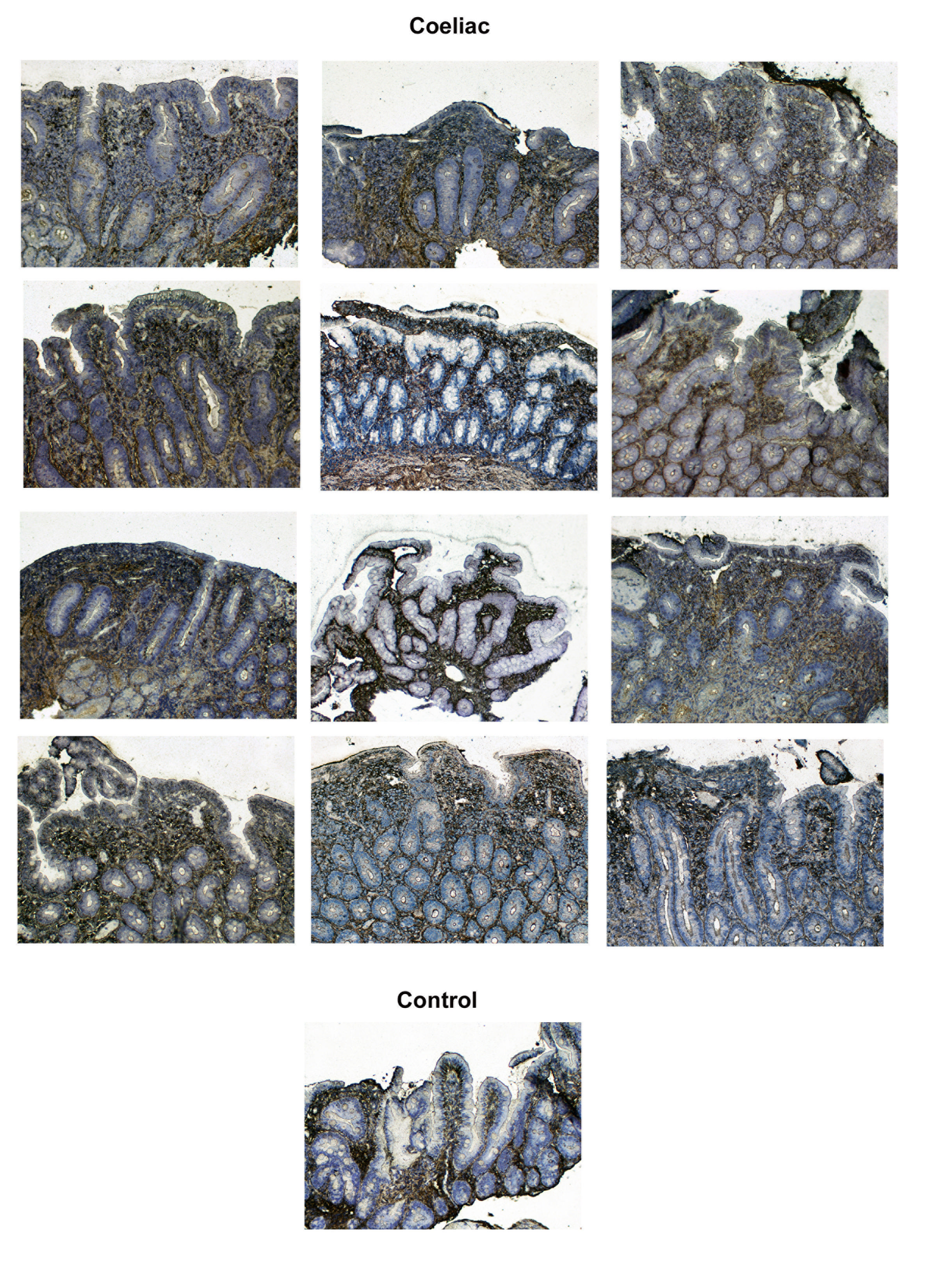

Supplement: Figure S5 — (TIF) [file pone.0106005.s005.tif]

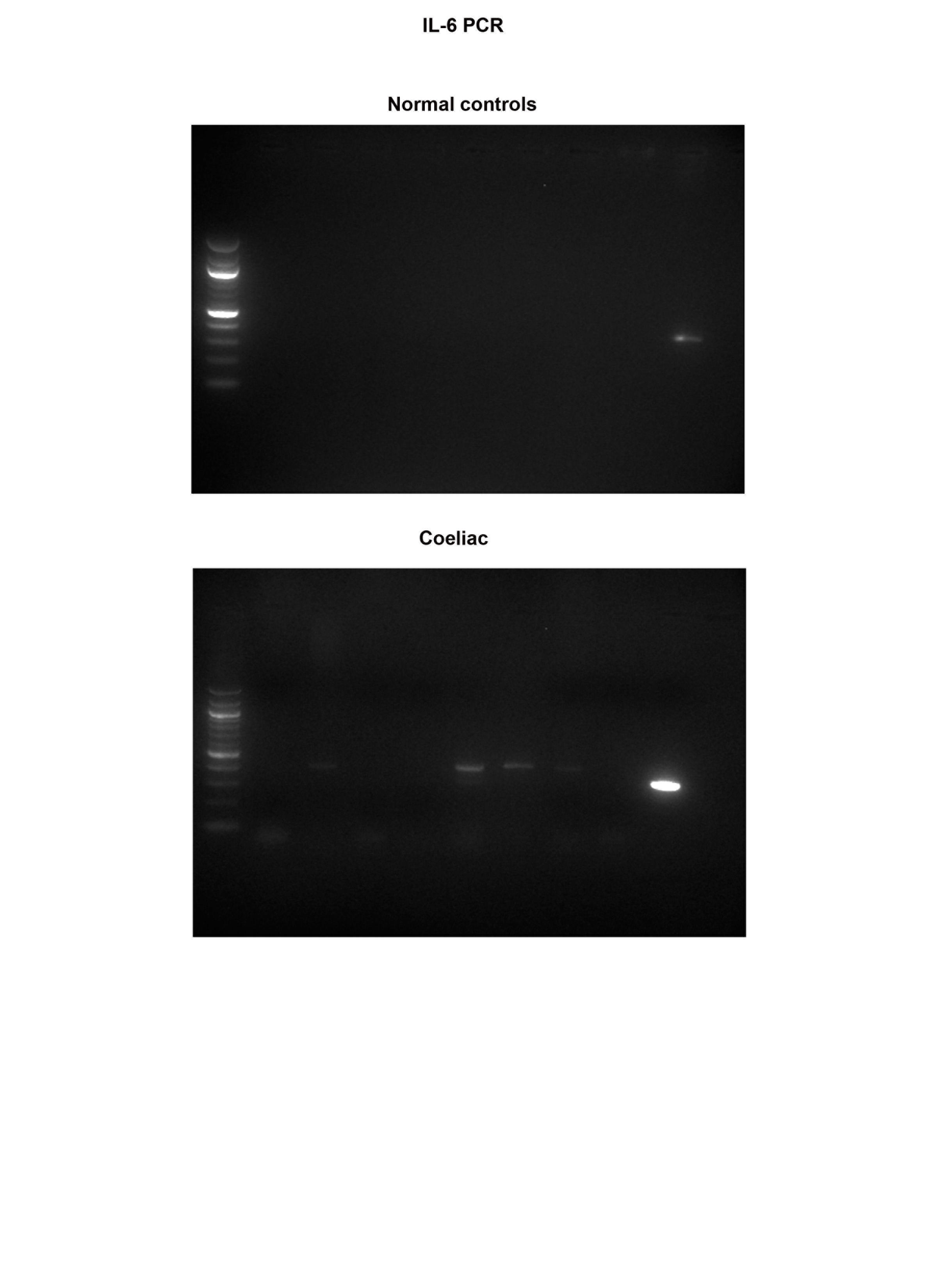

Supplement: Figure S6 — (TIF) [file pone.0106005.s006.tif]

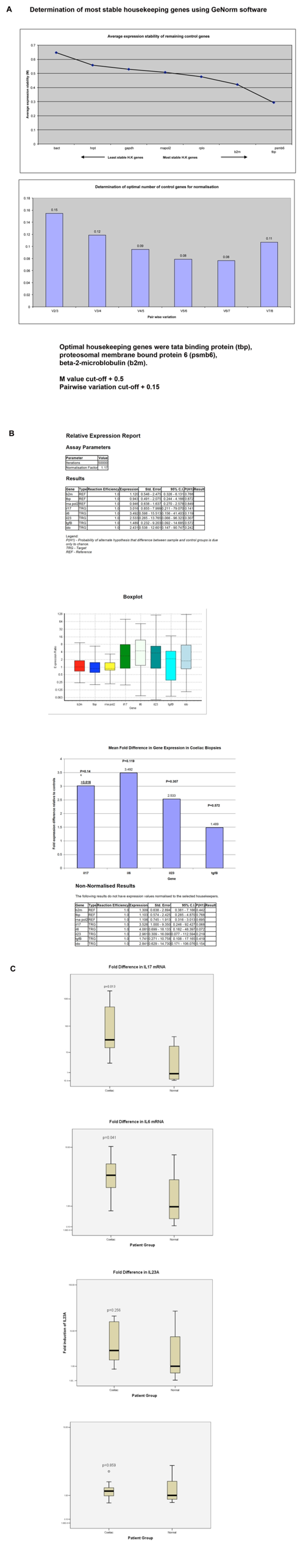

Supplement: Figure S7 — (TIF) [file pone.0106005.s007.tif]
